# First-Principles Simulations of Tip Enhanced Raman Scattering Reveal Active Role of Substrate on High-Resolution Images

**DOI:** 10.1021/acs.jpclett.3c01216

**Published:** 2023-07-24

**Authors:** Yair Litman, Franco P. Bonafé, Alaa Akkoush, Heiko Appel, Mariana Rossi

**Affiliations:** † Yusuf Hamied Department of Chemistry, University of Cambridge, Lensfield Road, Cambridge CB2 1EW, United Kingdom; ‡ MPI for the Structure and Dynamics of Matter, Luruper Chaussee 149, 22761 Hamburg, Germany; § Fritz Haber Institute of the Max Planck Society, Faradayweg 4−6, 14195 Berlin, Germany

## Abstract

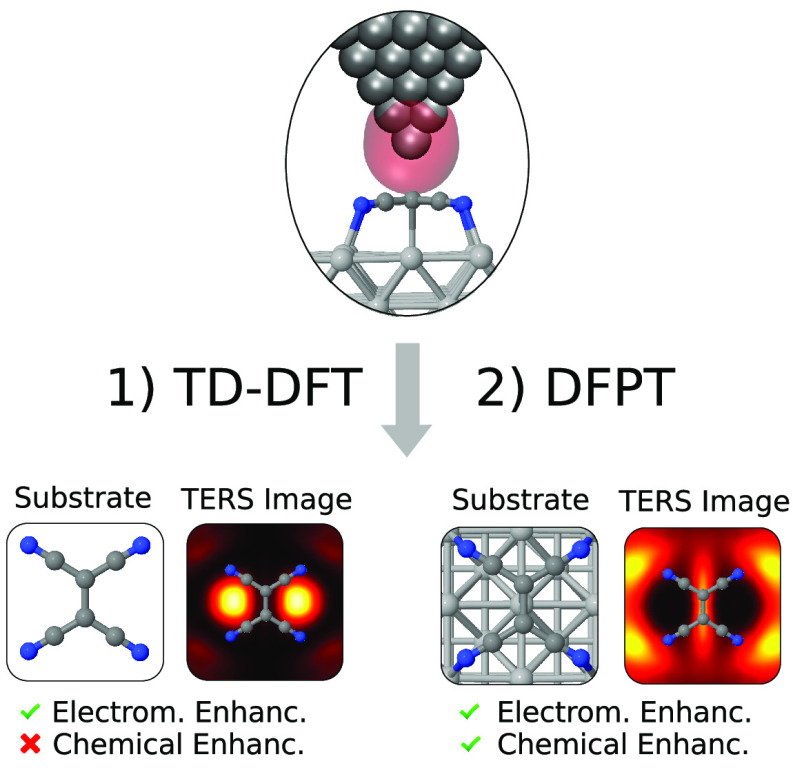

Tip-enhanced Raman scattering (TERS) has emerged as a powerful tool to obtain subnanometer spatial resolution fingerprints of atomic motion. Theoretical calculations that can simulate the Raman scattering process and provide an unambiguous interpretation of TERS images often rely on crude approximations of the local electric field. In this work, we present a novel and first-principles-based method to compute TERS images by combining Time Dependent Density Functional Theory (TD-DFT) and Density Functional Perturbation Theory (DFPT) to calculate Raman cross sections with realistic local fields. We present TERS results on free-standing benzene and C_60_ molecules, and on the TCNE molecule adsorbed on Ag(100). We demonstrate that chemical effects on chemisorbed molecules, often ignored in TERS simulations of larger systems, dramatically change the TERS images. This observation calls for the inclusion of chemical effects for predictive theory-experiment comparisons and an understanding of molecular motion at the nanoscale.

The atomic motion in materials and molecules drives structural changes and chemical reactions, thus, being of fundamental importance in areas as diverse as nanotechnology and biochemistry. Usually, vibrational modes are characterized indirectly through vibrational spectroscopy techniques that are incapable of resolving the motion of individual nuclei. Visualizing such motions with high spatial and temporal resolution is a long-sought goal that would allow an unambiguous understanding of certain physical and chemical processes.^1^ For individual molecules adsorbed on certain substrates, this visualization has been recently addressed by tip-enhanced Raman scattering (TERS).^2^

TERS spectroscopy is a powerful technique developed in the last two decades that seamlessly integrates the chemical specificity provided by Raman spectroscopy with the spatial sensitivity of scanning probe microscopy (SPM).^3−6^ Similar to other surface-enhanced techniques, the working principle of TERS relies on using the strongly localized plasmonic field produced at the tip apex by an external electromagnetic field, which enhances the Raman signal by several orders of magnitude.^7,8^ Unlike conventional spectroscopic techniques, where the spatial resolution is limited by the Rayleigh diffraction limit, near-field-enhanced techniques do not present this optical restriction. Indeed, depending on the shape of the tip apex and other experimental parameters, TERS setups can lead to subnanometer spatial resolution.^9^ TERS has been used to monitor catalytic processes at the nanoscale,^10^ study plasmon-driven chemical reactions,^11,12^ characterize 2D materials,^13−15^ and probe redox reactions at the solid/liquid interface.^16,17^ Arguably, the most impressive achievement obtained with TERS is the real space visualization of the vibrational modes of a single molecule, reported a few years ago.^2^

Regarding the physical processes underlying single-molecule TERS and the associated simulation protocols, there are still many points that need clarification. Besides the enhancement due to the strong localization of plasmonic electromagnetic fields (EM), there are three other possible enhancement mechanisms normally discussed in the literature and referred to as “chemical mechanisms”:^18^ (i) the enhancement due to the chemical interaction (e.g., orbital hybridization) between molecule and substrate or molecule and tip in the electronic ground state (chem-GS); (ii) the enhancement due to a resonance of the external field with a molecular electronic transition (chem-R); and (iii) the enhancement due to a charge transfer caused by the excitation-induced charge reorganization between the molecule and substrate or tip (chem-CT). While the EM mechanism is believed to be dominant in most cases, its relative importance is still under debate.^19−21^ For example, when the distance between a tip and a molecule is small enough to form a molecular point contact, a dramatic enhancement likely caused by chem-CT has been reported.^22−25^

Several methods to simulate TERS spectroscopy have recently been developed, with the aim of helping to interpret the increasing amount of experimental observations. There are methods based on phenomenological assumptions, which describe the localization of the near field by a bell-shaped function with a predefined width^2,26,27^ or which describe the local field by an oscillating dipole.^28,29^ These methods are relatively easy to implement and computationally inexpensive, but they are not *ab initio* and, thus, have limited predictive power. Other methods incorporate a realistic (classical) description of the near field,^30−32^ but the computational cost becomes prohibitively expensive for medium-sized systems, and a quantum description is restricted to a small region. All of these methods have provided valuable insights in specific situations. However, it is known that the exact atomistic structure of the tip influences the near field in nanoplasmonic junctions^33−35^ and that considering the electronic quantum effects in the description of nanoplasmonic fields is mandatory in certain conditions.^33,35^

In this work, we present a methodology that bridges the gap between some of the existing approaches. Our methodology incorporates a realistic description of the near field and retains a modest computational cost, making it applicable to adsorbed and large molecules. To achieve this, we employ density functional perturbation theory (DFPT) to compute the electric-field response of the electronic density that defines the nonresonant vibrational Raman cross sections but incorporate a realistic near-field distribution which we obtain from time-dependent density functional theory (TD-DFT) calculations of different atomistic tip geometries. In this way, we can capture the chem-GS and EM Raman enhancement mechanisms in our calculations at a cost comparable to phenomenological methods for medium and large systems but within a first-principles framework.

We consider a system composed of a molecule placed between a substrate and a metallic tip that lies at some position above the molecule (see Figure 1). If the distance between the tip and the substrate is larger than a few angstroms, there is no overlap of the corresponding charge densities, and therefore the interaction between the two components is dictated essentially by classical electrostatics.^36^ We study the effect induced on this system by a time-dependent transverse electromagnetic field, hereafter termed the external far field. Within the dipole approximation, this field is homogeneous. By formally separating the tip Hamiltonian from that of the rest of the system, we can write
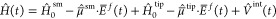
1where the labels “sm”, “tip”, and “int” refer to the substrate plus molecule subsystem, the tip subsystem, and the interaction between subsystems, respectively. *H*_0_ refers to the unperturbed Hamiltonians,  are the corresponding dipole operators, and *E̅*^*f*^(*t*) = (λ_*x*_***n*^**_*x*_ + λ_*y*_***n*^**_*y*_ + λ_*z*_***n*^**_*z*_) cos(ω_0_*t*), where λ_*x*,*y*,*z*_ are the electromagnetic field strengths, ω_0_ is the electromagnetic field frequency, and ***n*^**_*x*,*y*,*z*_ are unit vectors along each Cartesian direction. In the expression above, it is implicit that we work in a Coulomb gauge.

**Figure 1:**
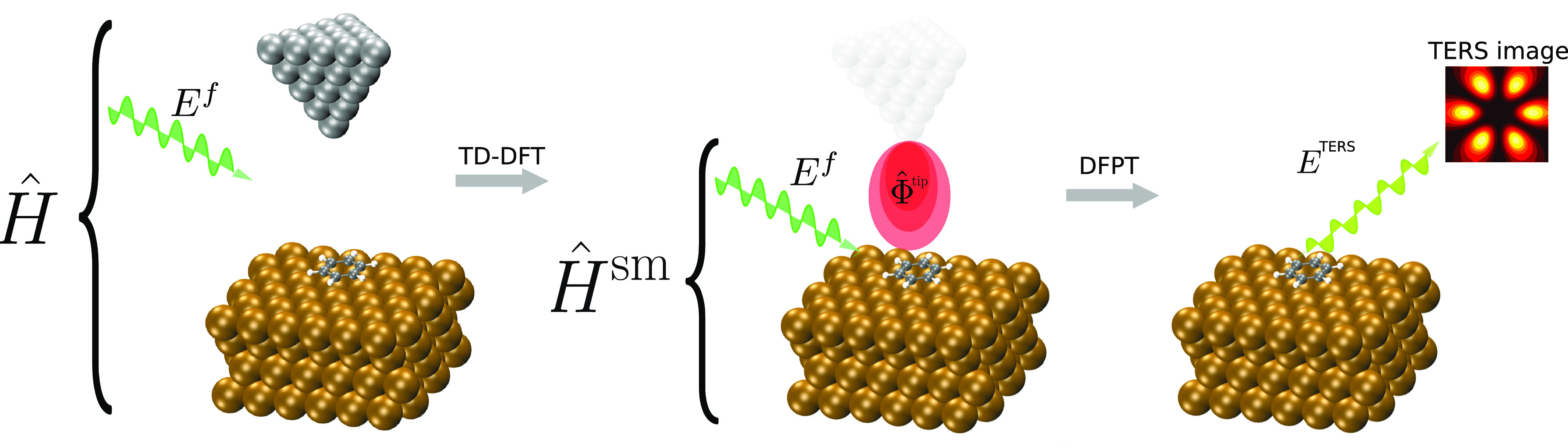
Schematic depiction of the proposed method. The full system and its corresponding full Hamiltonian, *Ĥ* (left), are approximated by that of the substrate–molecule subsystem, *Ĥ*^sm^ (center), which includes the perturbative terms associated with the external far field, *E*^*f*^, and the local field generated by the tip plasmonic oscillations, Φ̂^tip^, obtained from TD-DFT calculations. The calculation of TERS spectra proceeds through density-functional perturbation theory for the calculation of polarizability tensors.

To move forward, we make further assumptions. The first assumption is that the tip is not influenced by the presence of the molecule and substrate (justified by the previously assumed long distance between these components and a neutral molecule–substrate subsystem). This allows the calculation of the time-dependent electronic density ρ_tip_ by the real-time propagation of the Kohn–Sham states of the isolated tip under the influence of an external field in TD-DFT, assuming a dipolar light–matter coupling. Then, the (electrostatic) interaction between the tip and the rest of the system can be computed as
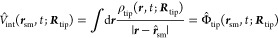
2where ***r***_sm_ refers to the positions of the electrons belonging to the substrate–molecule subsystem and ***R***_tip_ refers to the position of nuclei of the tip subsystem. In eq 2, we defined the time-dependent electrostatic potential of the tip, Φ̂_tip_ (often called Hartree potential), which is a central quantity for the current method. Indeed, under the current assumptions, the effect exerted on the substrate by the tip can be described by its Hartree potential. The ‘;’ symbol in eq 2 has been used to emphasize the parametric dependence of Φ̂_tip_ on the position and spatial arrangement of the nuclei in the tip, ***R***_tip_.

The second assumption is that the strength of the external far field is small, such that the response of the tip lies in the linear regime; i.e., one can perform a Taylor expansion of Φ̂_tip_ around zero-field strength (**λ** = 0) and truncate it at first order. The linear response regime was confirmed for the calculations presented throughout the paper (see Figure S4) and can be also verified in experimental setups. Then, considering that the system is at the ground state before an excitation by the laser field, Φ̂^tip^(*t* = 0) = Φ̂_GS_^tip^, and that responses are local in the frequency domain in the linear regime,^37^ we can write the substrate–molecule Hamiltonian in a particular Cartesian direction α as,
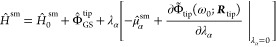
3where Φ̃_tip_(ω_0_; ***R***_tip_) denotes a time Fourier transform of Φ̂_tip_(*t*; ***R***_tip_) evaluated at ω_0_. In the last line, a perturbation of the substrate–molecule subsystem is neatly defined. The first term inside the square brackets describes the dipole interaction between the substrate with the homogeneous far field, while the second term describes the interaction with the local field generated by the tip. The latter term gives rise to the EM enhancement mechanism and the modified selection rules present in TERS spectroscopy. See a more detailed derivation of eq 3 in Section I of the Supporting Information (SI). Equation 3 is suitable to be treated within the *time-independent* DFPT in order to find the static polarizability of the molecule and substrate, α, which enables the calculation of the nonresonant Raman signal.^38^ In this work we calculate harmonic nonresonant Raman intensities, *I*^Raman^ as
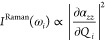
4where α_*zz*_ is the *zz* component of the polarizability tensor and *Q*_*i*_ and ω_*i*_ represent the eigenmode and eigenfrequency of the *i*-th vibrational normal mode, respectively. In this work, we consider exclusively the α_*zz*_ component since it is the direction normally regarded as the most relevant in TERS experiments^2,25^ and allows us to compare with previously reported spectra.^26,29^ However, the method can be used to describe any polarization dependence of the incoming and detected light by including other components of the polarizability tensor in the calculation of the Raman signal.^25,39,40^ Furthermore, it is possible to combine this approach with more sophisticated approximations of the Raman signal that can capture the anharmonicity of the vibrational modes, which could be relevant for more flexible molecules.^41,42^

In Figure 1, we show a schematic depiction of the proposed method. The electronic oscillations created by the external field generate an oscillating Hartree potential, Φ̂^tip^, whose gradient is the so-called local (longitudinal) electric field, and its maximum intensity is situated a few angstroms below the tip apex.^43^ The advantage of centering the approach on Φ̂^tip^ rather than the local field and its gradient is, besides its mathematical simplicity, the fact that all the terms in the multipolar expansion are automatically incorporated and no origin-dependence problems arise. All magnetic contributions are ignored as usually done for nonmagnetic materials.^44^ We note that the enhancement of the incident field is included, while the enhancement of the scattered field is ignored. To obtain the correct dependence of the enhanced Raman intensity with respect to the local field, the incorporation of dipole reradiation effects are required.^31,45^ Approximate corrections, based on the dressed tensor formalism, can be incorporated by choosing a coordinate origin and performing a Taylor expansion with respect to the incident fields.^44,46^ Within the formalism presented in this paper, a modified version of the latter approach would lead to an unphysical origin dependence. Thus, and similarly to most of the existing methods to simulate TERS images,^2,20,29,47^ the predicted signal intensity reported in this work follows a |*E*|^2^ dependence instead of the expected |*E*|^4^ for large tip-molecule distances.^18,31,48^

We start by analyzing the local Hartree potential generated by different Ag tip geometries. We considered tetrahedral tips with a one-atom apex (tip-A) and a three-atom apex (tip-B) as shown in Figure 2a,e, respectively.^22^ The fields Φ̃^tip^ were calculated using the Octopus code^49,50^ with the LDA exchange-correlation functional (see simulations details in Section II in the SI). The use of an arguably small model tip structure to study plasmonic near-field distributions from an atomistic first-principles perspective is justified by the fact that the plasmon peak of Ag clusters is well separated from the interband transitions even for small clusters.^51−53^Figure 2b,f shows the magnitude of Φ̃^tip^ as a function of the laser energy and distance from the tip apex. In both cases, the maximum Φ̃^tip^ is found at 1.4 Å below the tip apex and at 3.22 eV. The intensity of the potential decays to its half-value at 4 and at 5 Å below the tip apex for tip-A and tip-B, respectively. We analyzed larger tip sizes and verified that the overall shape of Φ̃^tip^ is not significantly altered and the plasmonic peak approaches the visible range in agreement with previous studies^51^ (Figure S3 in the SI). The two-dimensional cuts of Φ̃^tip^ for tip-A and tip-B, presented in the remaining panels of Figure 2, show that the field maximum is found exactly below the apex of tip-A and below the three atoms that constitute the tip apex for tip-B. Interestingly, at 6 Å below the tip apex, the shape of Φ̃^tip^ of the two models becomes indistinguishable, which suggests that, for substrate–tip distance greater than 6 Å, the fine details of the apex should be negligible in TERS imaging experiments. In passing, we note that at 4 Å below the tip apex the distribution of the local field resembles that of a 2D Gaussian function to some extent. However, a Gaussian profile can neither adequately describe the rapid change of intensity at the center of the distribution nor capture any radial asymmetry (see Section III in the SI).

**Figure 2:**
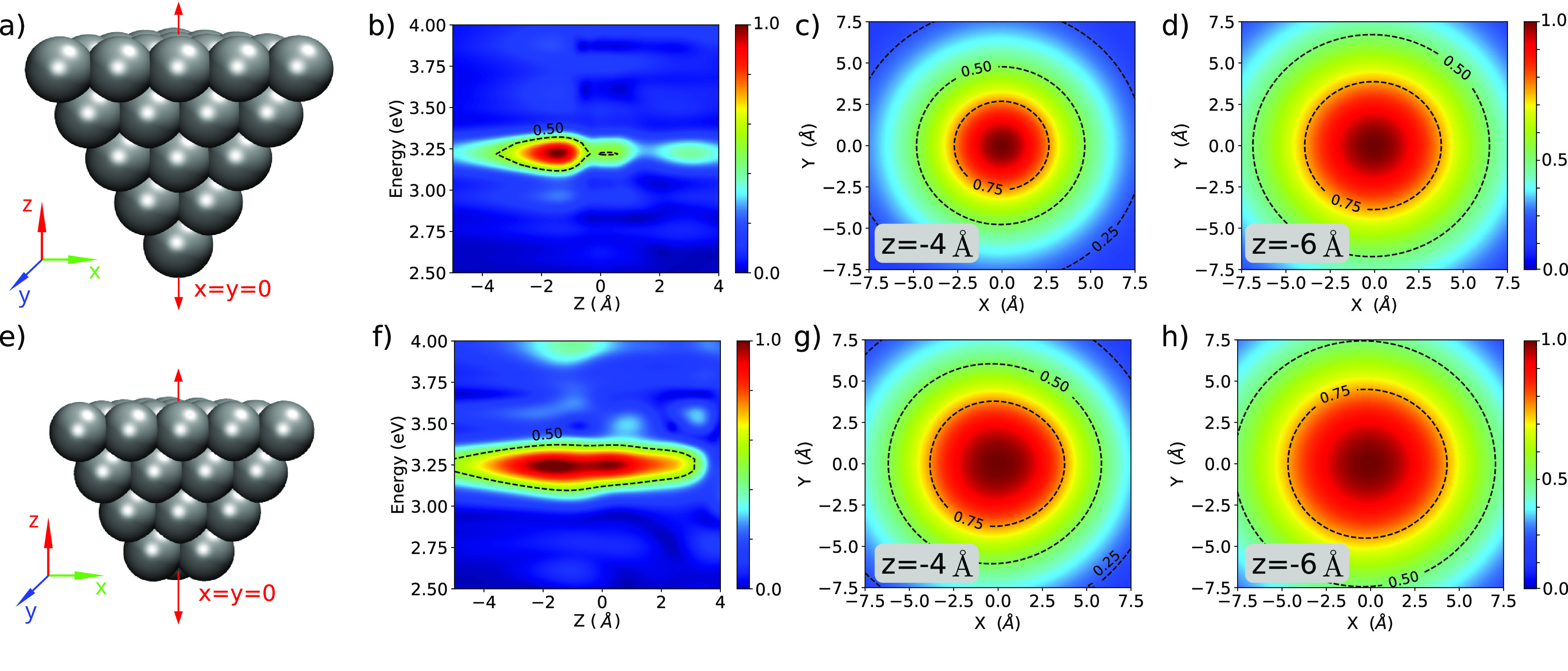
Energy and spatial dependence of the tip Hartree potential from TD-DFT simulations. Panel (a) shows the structure of the tip-A model. Panel (b) shows the normalized Φ̃^tip^ along the (*x* = 0, *y* = 0, *z*) line. Panels (c) and (d) show normalized two-dimensional cuts at 3.22 eV and *z* equal to 4 and 6 Å below the tip apex. Panels (e)–(h) are analogous to panels (a)–(d) for the tip-B model. In all plots the origin is defined at the center of the tip apex position.

We proceeded by computing TERS spectra for the free-standing benzene molecule. Benzene has been investigated several times as a proof-of-concept molecule^26,29^ and it allows us to compare the current method with others proposed in the literature. We calculated Raman intensities with the FHI-aims^54^ code and the LDA functional, where the DFPT implementation^55^ has been extended to include the local field as prescribed by eq 3 and to account for plasmonic terms in the electronic-density response of metallic clusters.^56^ We consider a benzene molecule in a flat orientation, as depicted in Figure 3a, and compute the TERS spectra for different tip–molecule distances, *d*, as shown in Figure 3b. In these calculations, tip-A was used, and its apex was aligned to the center of the benzene molecule. We remark that only the signal coming from the α_*zz*_ component of the polarizability tensor is shown. By analyzing the projected density of states of the benzene–tip system (see Section III in the SI) we concluded that, for distances larger than 3 Å, the assumption that there is no chemical interaction between the two subsystems is valid. Moreover, by analyzing the molecularly induced dipole at different tip–molecule relative positions, we verified that we are within the applicability realm of first-order perturbation theory at these molecule–tip distances (see Section II in the SI).

**Figure 3:**
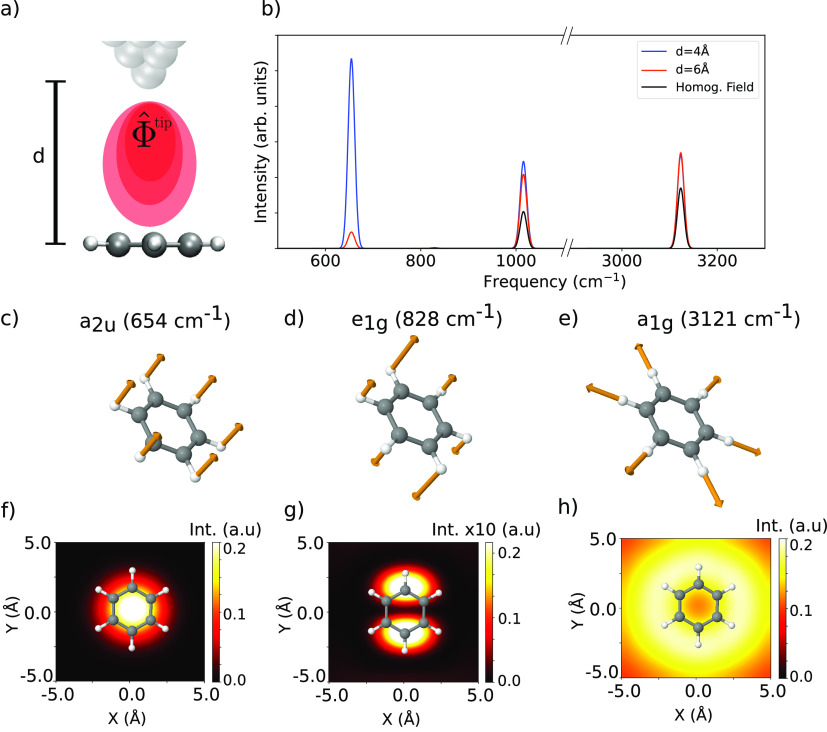
TERS simulation of gas-phase benzene from local-field DFPT calculations. (a) Sketch of the simulation setup. (b) Simulated harmonic TERS spectrum of the benzene molecule for different tip–molecule distances, *d*, compared to the homogeneous-field case. Only the signal coming from the α_*zz*_ component is shown for all cases. The observed enhancement is nonlinear with the molecule–tip distance since higher-order derivatives of the local potential start to contribute to the signal at the shortest distances.^44^ (c–e) Normal mode eigenvectors of selected vibrational modes with their respective symmetries and frequencies. (f–h) TERS images of the selected vibrational modes for a molecule–tip apex distance of 4 Å. See Figure S12 in the SI for the TERS image of the 1015 cm^–1^ peak.

The inhomogeneity of the local field induces changes in the TERS spectra in two distinctive ways compared to the standard (homogeneous field) Raman spectrum. On one hand, the intensity of the peaks at 1015 and 3121 cm^–1^ (*a*_1*g*_) is enhanced with respect to the homogeneous field case. On the other hand, the *a*_2*u*_ mode at 654 cm^–1^ which is Raman inactive becomes active in the TERS spectrum, which denotes a new selection rule arising from the spatial variation of the local field. In Figure 3 panels (c)–(e), we show the normal mode eigenvectors of selected vibrational modes, and in panels (f)–(h), their corresponding TERS images. The images were obtained by computing the TERS spectra at different lateral positions of the tip with respect to the molecule at a constant height of 4 Å. The intensities of the corresponding vibrational mode were then plotted in a 2D heat map. While the images of the modes located at 828 and 3121 cm^–1^ show distinctive patterns that are comparable to the ones obtained by other methods, the results for the mode located at 654 cm^–1^ obtained by our approach differs significantly.^26,29^ Although all of the methods would agree if the local field would be modeled by a given set of parameters, this example demonstrates the advantage of employing a parameter-free method, with easy-to-verify assumptions.

An interesting application of TERS spectroscopy is the determination of relative molecular orientations.^9,44,57^ Here, we evaluated the possibility of identifying the orientation of the C_60_ molecule using the current framework. In Figure 4, we report the TERS spectra of C_60_ in three different orientations. While we observe that the local field causes a nonuniform enhancement of peak intensities, the peaks that are active in the calculations with a homogeneous field, i.e., the H_*g*_ and A_*g*_ modes, are not sensitive to the specific molecular orientation. Conversely, some of the new peaks that emerge due to the local field, such as G_*g*_(5) and H_*g*_(2), present a more pronounced orientation dependence. Indeed, the corresponding TERS images, depicted in Figure 4, display characteristic patterns that could be used to identify the molecular orientation in sufficiently sensitive TERS experiments.

**Figure 4:**
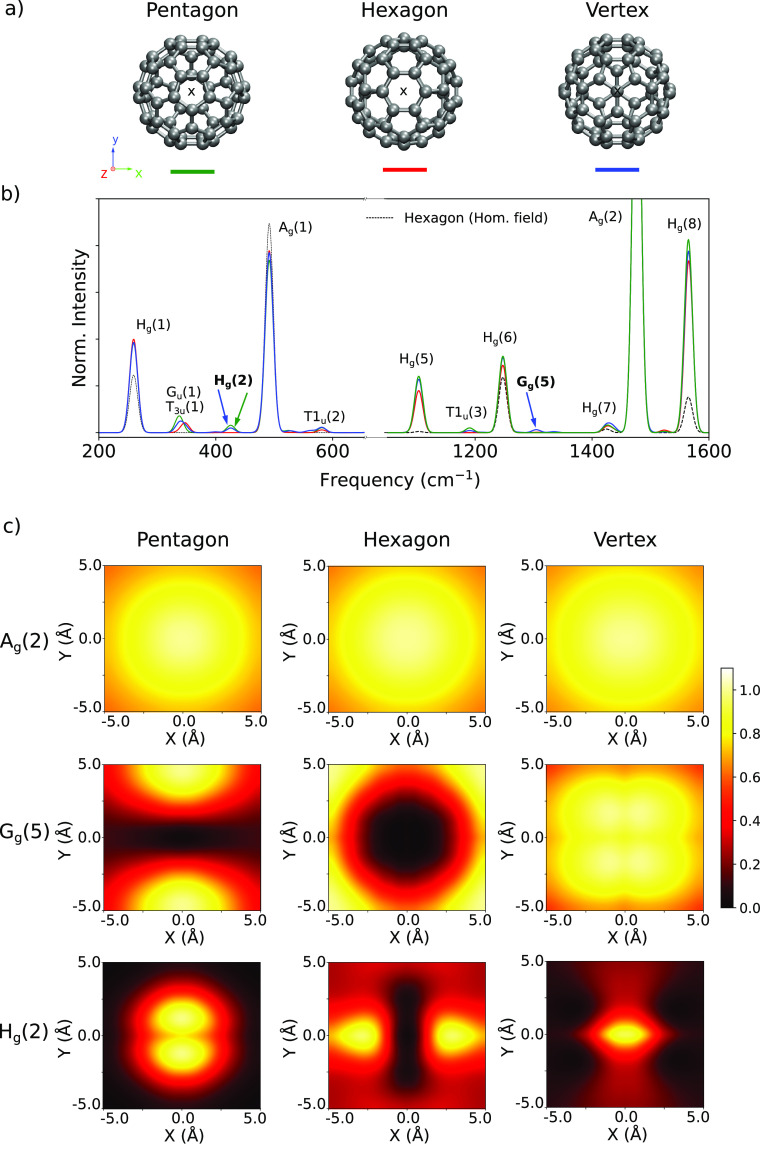
(a) Depiction of C_60_ molecular orientations. Pentagon (green), hexagon (red), and vertex (blue) refer to the molecular geometries with the corresponding face closest to the tip. (b) TERS spectra of the C_60_ molecule with the tip apex placed at 4 Å above the “X” mark in (a). The intensities of the spectra are normalized with respect to the intensity of the Ag(2) peak. (c) TERS images for the A_*g*_(2), G_*g*_(5), and H_*g*_(2) vibrational modes. In all cases, the intensities are normalized to the corresponding spectrum maximum.

Finally, we consider the tetracyanoethylene (TCNE) molecule as a representative of strong interaction with metallic substrates.^58^ TCNE is a strong electron acceptor due to the four cyano-group low-energy orbitals conjugated to the central C–C bond^59^ and has been investigated as a room temperature molecular magnet.^60^ To study the impact of chem-GS enhancements on TERS spectra, we consider three scenarios: (i) The TCNE molecule with its optimized geometry in the gas phase (TCNEgas), (ii) the molecule adsorbed on Ag(100) (TCNE@Ag(100)), and (iii) the molecule in the gas-phase but fixed at the adsorbed geometry (TCNEads). The Ag(100) surface was modeled by a 3-layer 4 × 4 cluster, and we employed the PBE functional in our DFPT calculations (see more details and convergence tests in Section II of the SI). The size limitation of the cluster models employed here is dictated only by technical issues regarding the implementation of DFPT for systems with fractional occupations, which can be easily overcome in the near future. In fact, we have recently computed TERS images of semiconducting systems containing nearly 200 atoms.^61^ Still, a few algorithmic hurdles need to be overcome to further increase the applicability of the method, as implemented in the FHI-aims code. For instance, the formulation of the DFPT response in real space and with the presence of a local field under periodic boundary conditions needs to be addressed.

TCNE is a planar molecule in the gas-phase. Upon adsorption with a flat orientation, the TCNE molecule arcs with the CN groups pointing toward the Ag atoms, and the nitrogen atoms coordinate Ag atoms that form a 3 × 3 square, as depicted in Figure 5a. The TCNE molecule becomes negatively charged upon adsorption, exhibiting an elongated central C–C bond.^59^ We estimated the molecular charge to be 0.6 e using a procedure described elsewhere.^62^ The TCNEgas, TCNEads, and TCNE@Ag(100) TERS spectra, calculated according to eq 4, are presented in Figure 5b with black, orange, and red curves, respectively. The TCNEgas spectrum presents three main peaks. The ones at 144 and 557 cm^–1^ correspond to out-of-plane modes, while the vibrations at 2239 cm^–1^ correspond to the in-plane CN stretching mode. The TCNEads spectrum also presents three major peaks at 207, 555, and 2119 cm^–1^, which correspond to analogous vibrational modes. However, due to the deformation of the molecular geometry, some of the vibrational frequencies are considerably red or blue-shifted. In addition, this spectrum presents several satellite peaks of relatively low intensity. The TCNE@Ag(100) spectrum is around 2 orders of magnitude more intense than the other spectra due to chem-GS enhancement. While the peak at 2127 cm^–1^ preserves the CN stretch character and is considerably enhanced, the modes at around 200 and 550 cm^–1^ mix with other normal modes and show a relatively smaller intensity enhancement. A new high-intensity peak appears at 1235 cm^–1^ and corresponds to the central C–C stretching mode.

**Figure 5:**
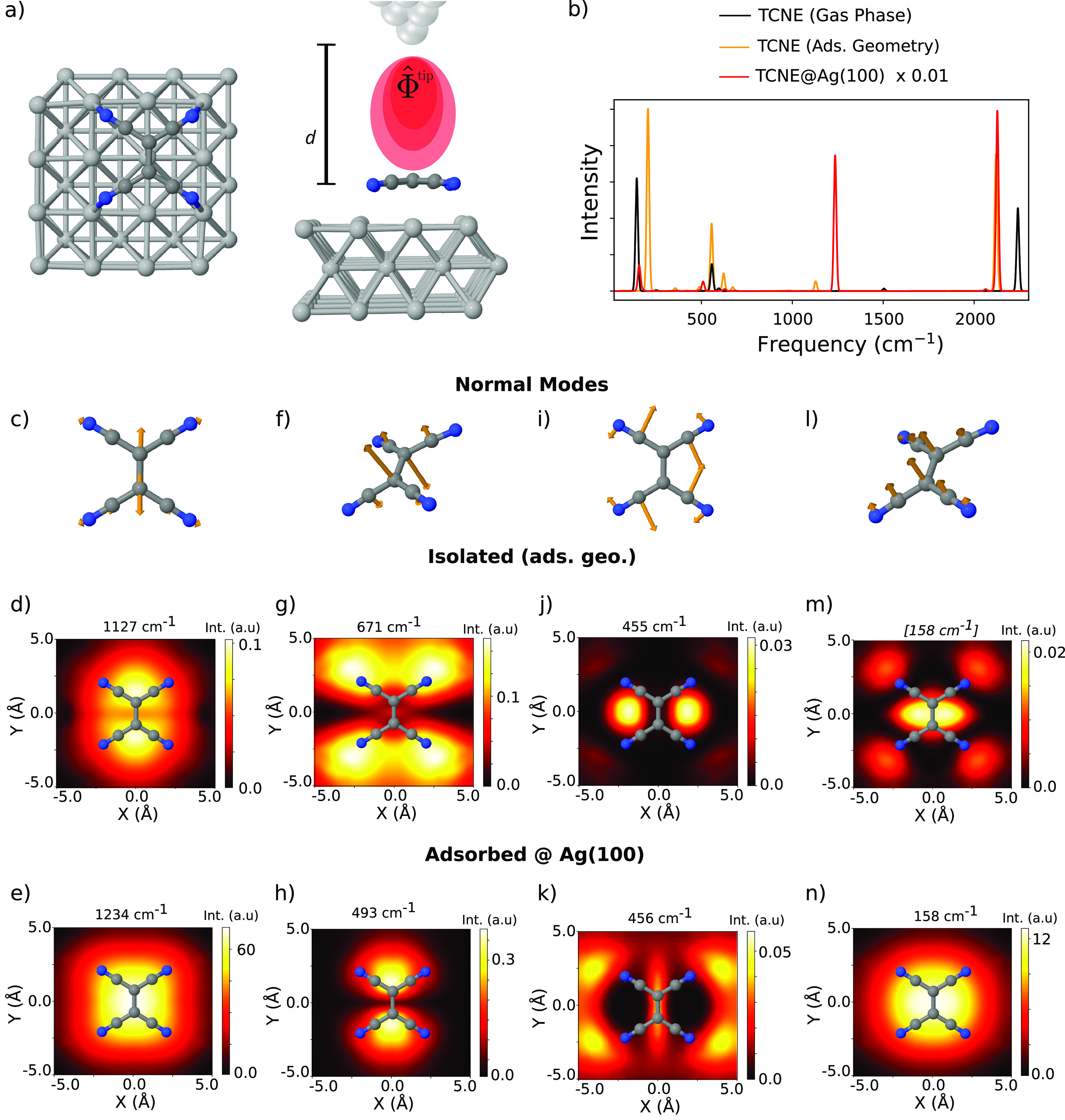
(a) Sketch of simulation setup of TCNE adsorbed on a Ag(100) cluster. (b) Simulated TERS image of TCNE in isolation (TCNEgas, black), of TCNE in isolation but at the adsorbed geometry (TCNEads, orange), and of TCNE at the Ag(100) cluster discussed in the text (TCNE@Ag(100), red). Tip apex was placed on the molecular center of mass at a distance of 4 Å. (c, f, i, and l) Normal mode displacement vectors of selected vibrational modes of TCNE@Ag(100). The surface has been deleted for clarity. (d, g, j, and m) TERS images of the depicted normal modes for TCNEads. (e, h, k, and n) TERS images of the depicted normal modes for TCNE@Ag(100). In all cases a molecule–apex distance of 4 Å was employed. Frequency within square brackets in panel (m) denotes the lack of an equivalent normal mode eigenvector in the TCNEads calculation.

In the remaining panels in Figure 5, we present TERS images for selected vibrational modes. To make a legit comparison and to isolate the effect caused by chem-GS enhancement, we only compare TCNEads with TCNE@Ag(100) (same molecular geometry), and in the evaluation of eq 4, we use the normal modes associated with the TCNE@Ag(100) structure. The TERS images of the central C–C stretching mode are shown in panels (d) and (e) and present comparable shapes with most of the Raman signal localized in the vicinity of the molecular center. However, the TCNEads image shows two clearly separated spots with the highest intensity at each side of the molecule along the central C–C bond axis. The intensity at the center of the molecule is relatively small, as shown in the 1D spectra. In panels (g), (h), (j), (k), (m), and (n) we present other vibrational modes that show TERS activity, including out-of-plane and in-plane molecular motions. The TERS images when including the Ag atoms are remarkably different even though we are considering the same geometry and nuclear displacements in the calculations. This observation proves that the symmetry of the normal modes does not exclusively determine TERS images and chem-GS effects can play a decisive role in determining the shape and intensity of the image. Moreover, neither a normal-mode analysis, a simple symmetry argument, nor a frequency comparison between TCNEads and TCNE@Ag(100) calculations seems to be able to predict, *a priori*, the impact of the chem-GS enhancement on the shape of the TERS images. We also verified that adding a negative charge to the TCNEads calculations does not reproduce the TCNE@Ag(100) results (see Figure S14 in the SI). This highlights once again the necessity of a first-principles calculation including the substrate.

In summary, we have presented a new first-principles method to compute TERS spectra and images that retains computational efficiency. The method does not rely on simplistic models for the tip geometry and its generated field and is able to capture EM and chem-GS types of Raman signal enhancement. It enables the calculation of TERS spectra and images at a substantially reduced computational cost. In fact, as shown in SI, Section V, we estimate a 4 orders of magnitude reduction in computational cost with respect to full real-time TD-DFT simulations.^63,64^ We presented results for three molecules: Two that physisorb on metallic substrates (benzene and C_60_) and one that chemisorbs (TCNE). For the former cases, we showed that the predicted TERS images differ from simplified approaches unless specific parameters are calibrated and confirmed that TERS spectroscopy can be used determine molecular orientations, even for highly symmetric molecules. For the latter, we showed that the chemical interaction between the molecule and the substrate leads to drastic changes in the TERS images, which reveal that the chemical enhancement shows atomic-scale variation.

The method proposed in this paper can be seamlessly coupled to *ab initio* (path integral) molecular dynamics simulations, to capture anharmonic and finite temperature (quantum) anharmonic effects.^41,65,66^ A calculation of the Raman intensities from a TD-DFT evaluation of the frequency-dependent polarizability tensors is also possible and would give access to resonant Raman scattering, thus capturing the chem-R enhancement mechanism.^63,67^ Moreover, by using methods with lower computational cost, such as density functional tight-binding,^68^ one could in principle converge the calculations with respect to the tip size.

The accuracy of the method we propose remains to be fully benchmarked, since a reference theoretical TERS calculation including all effects of light–matter coupling in the semiclassical limit^69,70^ has not yet been reported in the literature. Nevertheless, this method bridges an important gap in terms of accuracy and computational cost among existing approaches to TERS simulations, facilitating the interpretation of TERS experiments for realistic complex systems. We hope that the reported results motivate new single-molecule TERS experiments on inorganic–organic interfaces composed of chemisorbed molecules relevant to electronic and light-harvesting applications.^71−73^

## Data Availability

A tutorial to generate TERS images for the benzene molecule with the FHI-aims code with all the necessary input files is available at https://github.com/sabia-group/TERS_Tutorial.

## Supporting Information Available

Detailed derivation of eq 3, further computational details, and validation tests (PDF)
