# Supplementary material for: First-Principles Simulations of Tip Enhanced Raman Scattering Reveal Active Role of Substrate on High-Resolution Images
Source: J Phys Chem Lett. 2023 Jul 24;14(30):6850–9. doi: 10.1021/acs.jpclett.3c01216 (PMC10405274; doi:10.1021/acs.jpclett.3c01216)
Supplement: Supplementary file 1 — jz3c01216_si_001.pdf [file jz3c01216_si_001.pdf]

Supplemental Information: First-Principles Simulations of Tip Enhanced Raman Scattering Reveal Active Role of Substrate on High-Resolution Images

Yair Litman,[1,2,3,a)] Franco P. Bonafe,[2,3] Alaa Akkoush,[2,4] Heiko Appel,[2] and Mariana Rossi[2,4,b)]

[1)] *Yusuf Hamied Department of Chemistry, University of Cambridge, Lensfield Road, Cambridge, CB2 1EW,UK*

[2)] *MPI for the Structure and Dynamics of Matter, Luruper Chaussee 149, 22761 Hamburg, Germany*

[3)] *These authors contributed equally*

[4)] *Fritz Haber Institute of the Max Planck Society, Faradayweg 4–6, 14195 Berlin, Germany*

(Dated: 13 July 2023)

[a)]Electronic mail: yl899@cam.ac.uk

[b)]Electronic mail: mariana.rossi@mpsd.mpg.de

I. THEORY

A. Systems in the Presence of a Near Field

Consider a system perturbed by a transverse electromagnetic field. Under the long-wavelength approximation, the system is described by a Hamiltonian of the form

$$\hat{\boldsymbol{H}} = \hat{\boldsymbol{H}}_0 - \hat{\boldsymbol{\mu}} \cdot \boldsymbol{E}^n_{\perp}(t), \tag{1}$$

where $\hat{\boldsymbol{H}}_0$ refers to the unperturbed Hamiltonian, $\hat{\boldsymbol{\mu}}$ the system dipole operator, and $\boldsymbol{E}^n_{\perp}(t) = (\lambda_x \hat{\boldsymbol{n}}_x + \lambda_y \hat{\boldsymbol{n}}_y + \lambda_z \hat{\boldsymbol{n}}_z)\cos(\omega_0 t)$, where $\lambda_{x,y,z}$ are the electromagnetic field strengths and $\hat{\boldsymbol{n}}_{x,y,z}$ are unit vectors in each Cartesian direction. For clarity, in the following we consider the perturbation along a particular Cartesian direction α, since they are separable, but the derivation is easily generalized. Moreover, we consider, for the current derivation, that nuclei are clamped in space.

We shall consider that our system can be divided in two clearly distinguishable parts that we name 'sm' (i.e. substrate plus molecule) and tip which allows us to write the Hamiltonian of the full system as

$$\hat{H}_\alpha = \hat{H}_0^{\text{sm}} - \lambda_\alpha \hat{\mu}_\alpha^{\text{sm}} \cos(\omega_0 t) + \hat{H}_0^{\text{tip}} - \lambda_\alpha \hat{\mu}_\alpha^{\text{tip}} \cos(\omega_0 t) + \hat{H}^{\text{int}}, \tag{2}$$

where the label 'int' refers to 'sm-tip' interaction.

We will assume that the tip is not influenced by the presence of the molecule, which allow us to write a tip Hamiltonian,

$$\hat{H}_\alpha^{\text{tip}} = \hat{H}_0^{\text{tip}} - \lambda_\alpha \hat{\mu}_\alpha^{\text{tip}} \cos(\omega_0 t), \tag{3}$$

and obtain the time-dependent wave function $\left|\Psi_\alpha^{\text{tip}}(\boldsymbol{r}^{\text{tip}}, t)\right\rangle$ without reference to the molecule subsystem. Moreover, the lack of influence of the molecule on the tip implies that the interaction is purely electrostatic (charge transfer or dispersion are not possible). This approximation is reasonable for neutral molecules and for all but very small tip-molecule distances. Under the previous assumptions the interaction Hamiltonian gets transformed in an effective interaction Hamiltonian, $\hat{H}^{\text{int,eff}}$ defined as the following expectation value

$$
\begin{aligned}
\left\langle \Psi_\alpha^{\mathrm{tip}}(\boldsymbol{r}^{\mathrm{tip}}, t; \boldsymbol{R}^{\mathrm{tip}}) \right| \hat{H}^{\mathrm{int}}(\boldsymbol{r}^{\mathrm{tip}}, \boldsymbol{r}^{\mathrm{sm}}, t) \left| \Psi_\alpha^{\mathrm{tip}}(\boldsymbol{r}^{\mathrm{tip}}, t; \boldsymbol{R}^{\mathrm{tip}}) \right\rangle &= \hat{H}_\alpha^{\mathrm{int}}(\boldsymbol{r}^{\mathrm{sm}}, t; \boldsymbol{R}^{\mathrm{tip}}) \\
&= e \int d\boldsymbol{r} \frac{\rho_\alpha^{\mathrm{tip}}(\boldsymbol{r}, t; \boldsymbol{R}^{\mathrm{tip}})}{|\hat{\boldsymbol{r}}^{\mathrm{sm}} - \boldsymbol{r}|} \\
&= e \hat{\Phi}_\alpha^{\mathrm{tip}}(\boldsymbol{r}^{\mathrm{sm}}, t; \boldsymbol{R}^{\mathrm{tip}})
\end{aligned} \tag{4}
$$

where $\boldsymbol{r}_{\mathrm{sm}}$ ($\boldsymbol{r}_{\mathrm{tip}}$) refers to the position of electrons that belong to the substrate-molecule (tip) subsystem, $\boldsymbol{R}_{\mathrm{tip}}$ refers to position of nuclei that belong to the tip subsystem, and the ';' symbol has been used to emphasis the parametric dependence. $\hat{\Phi}_\alpha^{\mathrm{tip}}(\boldsymbol{r}, t; \boldsymbol{R}^{\mathrm{tip}})$ is the time-dependent Hartree potential generated by the tip upon interaction with the α component of the far field, and it depends on the position operator and parametrically on the coordinates of the tip. It represents the key quantity of the new approach. For reasons that will become clear later, we shall expand the time-dependent Hartree potential around zero field strength,

$$
\begin{aligned}
\hat{\Phi}_\alpha^{\mathrm{tip}}(\boldsymbol{r}, t; \boldsymbol{R}^{\mathrm{tip}}) &= \hat{\Phi}_\alpha^{\mathrm{tip}}(\boldsymbol{r}, t; \boldsymbol{R}^{\mathrm{tip}})|_{\lambda_\alpha=0} + \lambda_\alpha \left. \frac{\partial \hat{\Phi}_\alpha^{\mathrm{tip}}(\boldsymbol{r}, t; \boldsymbol{R}^{\mathrm{tip}})}{\partial \lambda_\alpha} \right|_{\lambda_\alpha=0} + O(\lambda^2) \\
&\approx \hat{\Phi}_{\mathrm{GS}}^{\mathrm{tip}}(\boldsymbol{r}; \boldsymbol{R}^{\mathrm{tip}}) + \lambda_\alpha \left. \frac{\partial \hat{\Phi}_\alpha^{\mathrm{tip}}(\boldsymbol{r}, t; \boldsymbol{R}^{\mathrm{tip}})}{\partial \lambda_\alpha} \right|_{\lambda_\alpha=0}
\end{aligned} \tag{5}
$$

where we used the fact that in the absence of an external field, the tip is in the electronic ground state (GS). Since we consider a continuous laser and using the fact that linear responses are local in the frequency domain (i.e. the density oscillates predominantly at the frequency of the external field), we can conveniently write

$$
\left. \frac{\partial \hat{\Phi}_\alpha^{\mathrm{tip}}(\hat{\boldsymbol{r}}, t; \boldsymbol{R}^{\mathrm{tip}})}{\partial \lambda_\alpha} \right|_{\lambda_\alpha=0} = \Re \left[\left. \frac{\partial \tilde{\Phi}_\alpha^{\mathrm{tip}}(\boldsymbol{r}, \omega_0; \boldsymbol{R}^{\mathrm{tip}})}{\partial \lambda_\alpha} \right|_{\lambda_\alpha=0} \right] \cos(\omega_0 t) \tag{6}
$$

where $\tilde{\Phi}^{\mathrm{tip}}$ denotes the Fourier transform the tip Hartree potential. We note that since we are interested in the simulation of time-independent TERS spectroscopy which represents a steady-state excitation, we have the freedom to arbitrarily define the initial time and therefore keep either the real or imaginary part in Eq. 6. However, for later consistency, we have chosen the former and will use this fact later on.

By introducing Eq. 4, 5 and 6 into Eq. 2 and collecting all the terms that depend on the molecular degrees of freedom and are linear on λ, we arrive at the following expression for

the molecular Hamiltonian

$$
\begin{aligned}
\hat{H}_{\alpha}^{\mathrm{sm}} &= \hat{H}_{0}^{\mathrm{sm}} + \hat{H}_{\alpha}^{\mathrm{int}}(\hat{\boldsymbol{r}}^{\mathrm{sm}}, t; \boldsymbol{R}^{\mathrm{tip}}) - \lambda_{\alpha}\mu_{\alpha}^{\mathrm{sm}}\cos(\omega_0 t) \\
&= \hat{H}_{0}^{\mathrm{sm}} + \hat{\Phi}_{\mathrm{GS}}^{\mathrm{tip}}(\boldsymbol{r}; \boldsymbol{R}^{\mathrm{tip}}) + \lambda_{\alpha}\left\{ -\mu_{\alpha}^{\mathrm{sm}} + \Re\left[\left.\frac{\partial \tilde{\Phi}_{\alpha}^{\mathrm{tip}}(\boldsymbol{r}, \omega_0; \boldsymbol{R}^{\mathrm{tip}})}{\partial \lambda_{\alpha}}\right|_{\lambda_{\alpha}=0} \right] \right\} \cos(\omega_0 t)
\end{aligned} \tag{7}
$$

We remark that the previous expression is origin independent and that Eq. 3 presented in the main text represents its *time-independent* version.

B. Time Independent Density Functional Perturbation Theory

In the framework of Kohn–Sham (KS) DFT, the total energy can be expressed as functional of the electron density, ρ, as

$$
E^{(0)}[\rho] = T_s[\rho] + E_{\mathrm{ext}}[\rho] + E_{\mathrm{H}}[\rho] + E_{\mathrm{xc}}[\rho] + E_{\mathrm{nn}}[\rho] \tag{8}
$$

where T_s, E_{ext}, E_{H}, E_{xc}, and E_{nn} are the kinetic energy of non-interacting electrons, the external energy due to the the electron-nuclei electrostatic attraction, the Hartree energy, the exchange-correlation energy, and the nuclei-nuclei electrostatic interaction energy , respectively.

The ground-state total energy is obtained variationally under the constraint that the number of electrons is constant which leads to the Kohn–Sham single-particle equations

$$
\hat{h}_{\mathrm{KS}}\psi_p = \epsilon_p \psi_p, \tag{9}
$$

where ψ_p and ϵ_p are the KS single particle states and energies, and

$$
\hat{h}_{\mathrm{KS}} = \hat{t}_{\mathrm{s}} + \hat{\nu}_{\mathrm{ext}} + \hat{\nu}_{\mathrm{H}} + \hat{\nu}_{\mathrm{xc}} \tag{10}
$$

is the KS single particle Hamiltonian. All the terms on the right hand-side of the previous equation are single-particle operators which represent the kinetic energy, $\hat{t}_{\mathrm{s}}$, the external potential, $\hat{\nu}_{\mathrm{ext}}$, the Hartree potential, $\hat{\nu}_{\mathrm{H}}$ and the exchange-correlation functional, $\hat{\nu}_{\mathrm{xc}}$. The ground-state electronic density is computed by $\rho_{\mathrm{GS}}(\mathbf{r}) = \sum_p f(\epsilon_p)|\psi_p(\mathbf{r})|^2$ where f is the occupation function.

We now consider a perturbation due to an external electromagnetic field, in the framework of *time independent* perturbation theory. The energy functional gets an extra term, $E_{\mathrm{E}}[\rho]$, and the perturbed KS single-particle Hamiltonian can be expressed as

$$\hat{h}_{\mathrm{KS}}(\epsilon_\alpha) = \hat{h}^{(0)}_{\mathrm{KS}} + \hat{h}^{(1)}_{\mathrm{KS}}\lambda_\alpha + \dots \tag{11}$$

where λ_α is the strength of the external field along the direction α, $\hat{h}^{(1)}_{\mathrm{KS}}$ is the first order response of the Hamiltonian operator given by

$$\hat{h}^{(1)}_{\mathrm{KS}} = +\hat{\nu}^{(1)}_{\mathrm{ext}} + \hat{\nu}^{(1)}_{\mathrm{H}} + \hat{\nu}^{(1)}_{\mathrm{xc}} + \hat{\nu}_{\mathrm{E}}, \tag{12}$$

and $\hat{\nu}^{(1)}_{\mathrm{E}}$ is the (still not specified) coupling operator between the system and the external electromagnetic field. By introducing analogous expansions for the single-particle states ($\psi_p = \psi^{(0)}_p + \psi^{(1)}_p\epsilon_\alpha + \dots$) and their eigenenergies ($\epsilon_p = \epsilon^{(0)}_p + \epsilon^{(1)}_p\lambda_\alpha + \dots$) , one reaches the well-known Sternheimer equation which reads

$$(\hat{h}^{(0)}_{\mathrm{KS}} - \epsilon_0)\psi^{(1)}_p = -(\hat{h}^{(1)}_{\mathrm{KS}} - \epsilon_1)\psi^{(0)}_p. \tag{13}$$

The solution of the Sternheimer equation gives direct access to the density response defined as

$$\rho^{(1)}_\alpha(\boldsymbol{r}) = \frac{\partial\rho(\boldsymbol{r})}{\partial\lambda_\alpha} = \sum_p f(\epsilon_p)[\psi^{(1)}_p(\boldsymbol{r})\psi^{(0)}_p(\boldsymbol{r}) + \psi^{(0)}_p(\boldsymbol{r})\psi^{(1)}_p(\boldsymbol{r})], \tag{14}$$

and allows for the calculation of the induced dipole as

$$\mu^{\mathrm{ind}}_\alpha = \sum_{\beta=1}^{3}\lambda_\beta\int d\boldsymbol{r}\rho^{(1)}_\beta(\boldsymbol{r})r_\alpha = \sum_\beta \lambda_\beta\alpha_{\alpha\beta}, \tag{15}$$

in which we identify the components of the polarizability tensor as

$$\alpha_{\alpha\beta} = \frac{\partial\mu^{\mathrm{ind}}_\alpha}{\partial\lambda_\beta} = \int d\boldsymbol{r}\rho^{(1)}_\beta(\boldsymbol{r})r_\alpha. \tag{16}$$

It is central to the following developments that we arrived to Eq. 16 without determining the specific nature of the perturbation, $\hat{\nu}_{\mathrm{E}}$, besides the assumption that it is weak enough to allow the omission of electrical non-linear effects. We now consider different suitable forms for $\hat{\nu}_E$.

1. *Homogeneous Field Perturbation*

In most of the Raman experiments the frequency of the (monochromatic) external electromagnetic field falls in the visible range. In these cases, the field remains approximately constant across the molecular dimensions and it is valid to apply the long-wavelength approximation. Thus, for this homogeneous and time-independent field, E_{E} is expressed as

$$E_E[\rho] = -\boldsymbol{\lambda} \cdot \int d\boldsymbol{r} \rho(\boldsymbol{r}) \boldsymbol{r}, \tag{17}$$

and the corresponding single-particle operator becomes

$$\hat{\nu}_{\mathrm{E}} = -\boldsymbol{r}. \tag{18}$$

2. *Linear Field*

The next step towards the inclusion of spatial-dependent fields is to consider a field with a non-vanishing gradient. The extra energy term becomes

$$E_E[\rho] = -\sum_{\alpha} \lambda_\alpha \left[\int d\boldsymbol{r} \rho(\boldsymbol{r}) r_\alpha - \sum_{\beta} \frac{1}{2} \int d\boldsymbol{r} \frac{\partial \lambda_\alpha}{\partial r_\beta} \rho(\boldsymbol{r}) r_\alpha r_\beta \right], \tag{19}$$

and the single-particle operator is given by

$$\hat{h}^{(1)}_{\mathrm{KS}} = \hat{\nu}^{(1)}_{\mathrm{ext}} + \hat{\nu}^{(1)}_{\mathrm{H}} + \hat{\nu}^{(1)}_{\mathrm{xc}} - r_\alpha - \sum_{\beta} \frac{1}{2} \frac{\partial \lambda_\alpha}{\partial r_\beta} r_\alpha r_\beta. \tag{20}$$

It is clear to see that this approach becomes impractical rather quickly if one wants to consider higher-order derivatives. Moreover, if the field is not strictly linear, the previous expression becomes origin dependent since the value of $\frac{\partial \lambda_\alpha}{\partial r_\beta}$ is position dependent.

3. *Inclusion of Near Fields*

We now consider that the Hamiltonian is given by Eq. 7, but in its *time independent* form. The perturbation up to the first order is given by

$$-\mu_\alpha + \Re \left[\frac{\partial \tilde{\Phi}^{\mathrm{tip}}_\alpha(\boldsymbol{r}, \omega_0; \boldsymbol{R}^{\mathrm{tip}})}{\partial \lambda_\alpha} \bigg|_{\lambda_\alpha = 0} \right] \tag{21}$$

and the energy gain can be expressed as

$$E_E[\rho] = \sum_\alpha \lambda_\alpha \left[- \int d\boldsymbol{r}\rho(\boldsymbol{r}) r_\alpha + \int d\boldsymbol{r}\rho(\boldsymbol{r}) \Re \left[\frac{\partial \tilde{\Phi}^{\text{tip}}_\alpha(\boldsymbol{r}, \omega_0; \boldsymbol{R}^{\text{tip}})}{\partial \lambda_\alpha} \bigg|_{\lambda_\alpha=0} \right] \right]. \tag{22}$$

The first order response of the Hamiltonian operator is given by

$$\hat{h}^{(1)}_{\text{KS}} = \hat{\nu}^{(1)}_{\text{ext}} + \hat{\nu}^{(1)}_{\text{H}} + \hat{\nu}^{(1)}_{\text{xc}} - r_\alpha + \Re \left[\frac{\partial \tilde{\Phi}^{\text{tip}}_\alpha(\boldsymbol{r}, \omega_0; \boldsymbol{R}^{\text{tip}})}{\partial \lambda_\alpha} \bigg|_{\lambda_\alpha=0} \right]. \tag{23}$$

We note that in this case, the ground-state KS Hamiltonian $\hat{h}^0_{\text{KS}}$ also gets modified by the addition of the $\Phi^{\text{tip}}_{\text{GS}}$ term.

Summary of approximations and assumptions in the derivation of Eq 7

What follows is a point-by-point summary of the approximation and assumptions employed in the previous section:

- Eq. 1: Dipole coupling between the system and the far-field.
- Eq. 2: The interaction between the tip and substrate is dictated by classical electrostatics.
- Eq. 3: the tip is not influenced by the presence of the substrate.
- Eq. 5: the external far field strength is small enough, such that the response of the tip lies in the linear regime.
- Eq. 11: the external far field strength is small enough, such that the response of the substrate is linear with respect of the local field strength.

II. METHODS

A. DFT and DFPT calculations

All the electronic DFT and DFPT calculations were carried out using FHI-aims code[1] with the 'light' default settings for numerical grids and basis sets. The calculations for the benzene molecule were carried out with LDA exchange correlation functional as parameterized by Perdew and Wang[2]. The calculations for the TCNE molecule in the gas phase

and adsorbed on Ag(100) were carried out with the PBEexchange-correlation functional instead, to have a better description of the charge transfer between the metal substrate and the molecule. A pair-wise van der Waals correction specifically tailored for hybrid organic-inorganic systems was used in all the calculation containing the Ag(100) surface[3]. The Ag(100) cluster was modeled by a 3-layer 4×4 cluster where only the first two top layers were allowed to relax and the atoms on the bottom one were kept fixed in their bulk positions. Selected calculations were repeated with a 3-layers 5×6 cluster model, see discussion in section IV. The cluster models were created using the atomic simulation environment (ASE)[4] using a 4.157 Å lattice constant for Ag. The geometries were relaxed within FHI-aims up to a maximum residual force component per atom of 0.005 eV/A. In Fig. S1, the minimum energy structures of the TCNE molecule adsorbed in the cluster models are depicted.

We compared the projected density of stated (PDOS) of the cluster calculations with the ones obtained from periodic calculations using a 3-layer Ag 3×4 slab and a k-grid of $4 \times 4 \times 1$. As shown in Fig. S2, the PDOS of TCNE on the cluster model is in reasonable agreement with the periodic calculations and markedly different from the gas phase PDOS.

The calculation of the Raman intensities were performed by the evaluation of Eq. 4 in the main text by a symmetric finite difference approach. All the atoms in the molecule were displaced by 0.002 Å along all Cartesian directions. All the presented TERS images were computed with 0.5 Å $\times$ 0.5 Å resolution after verifying with the benzene molecule that using a 0.25 Å$\times$ 0.25 Å resolution does not result in significant changes on the image.

B. TD-DFT calculations

The real-time TDDFT calculations were carried out with the Octopus code[5,6] , employing the adiabatic local density approximation (ALDA) to describe exchange-correlation effects unless stated otherwise. The field perturbation was introduced by a Dirac delta perturbation in time, also known as '*kick*', at the initial time with field strength k, $\boldsymbol{E} = -\hbar k/e\delta(t)$, which causes the initial wavefunction to instantaneously acquire a phase factor. We utilized a time step of 0.0065 atomic units of time to integrate the time-dependent Kohn-Sham equations of motion and run the simulations for 30000 steps, saving the Hartree potential every 10 steps in cube file format. We employed field strengths between $5{\times}10^{-4}$ Å^{-1} and $1.5{\times}10^{-3}$

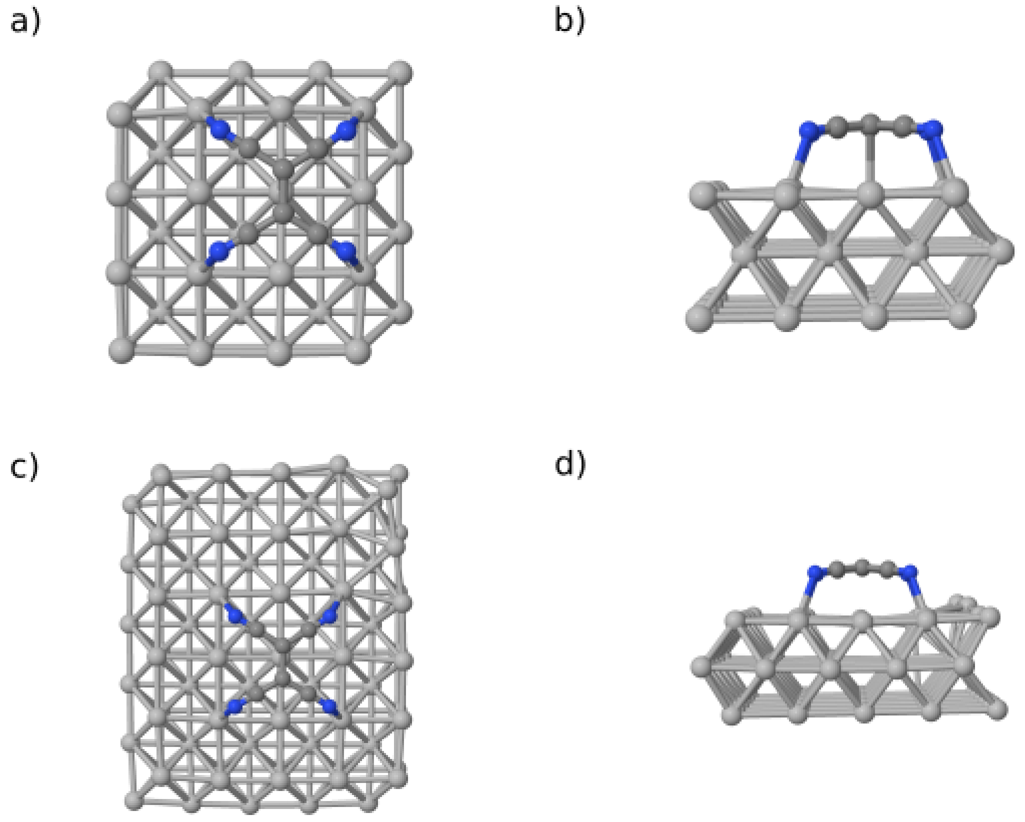

FIG. S1: Top and lateral view of 4 x 4 (top) and 5 x 6 (bottom) cluster models employed to study the adsorption of the TCNE molecule adsorbed at Ag(100).

$Å^{-1}$, and verified to be within the linear-response regime (see Fig. S4). The derivative of $\Phi^{\rm tip}$ with respect to the field strength was obtained as

$$\begin{aligned}\frac{\partial\tilde{\Phi}^{\rm tip}(\boldsymbol{r},\omega)}{\partial\lambda_{\gamma}^{\rm far}} &= \frac{\int dt e^{i\omega t}\phi^{\rm tip}(\boldsymbol{r},t)}{\int dt e^{i\omega t}\hbar k/e\delta(t)}, \\ &= \frac{\int dt e^{i\omega t}\phi^{\rm tip}(\boldsymbol{r},t)}{\hbar k/e}.\end{aligned} \tag{24}$$

In all the TERS calculations the plasmonic frequency was chosen, i.e. $\omega = 3.22eV$.

III. VALIDATION TEST

In Fig. S3, we show $\tilde{\Phi}^{\rm tip}$ obtained for different tip sizes. The overall shape of $\tilde{\Phi}^{\rm tip}$ below the tip apex is not significantly modified. However, the plasmonic peak approaches the visible range in agreement with previous studies[7].

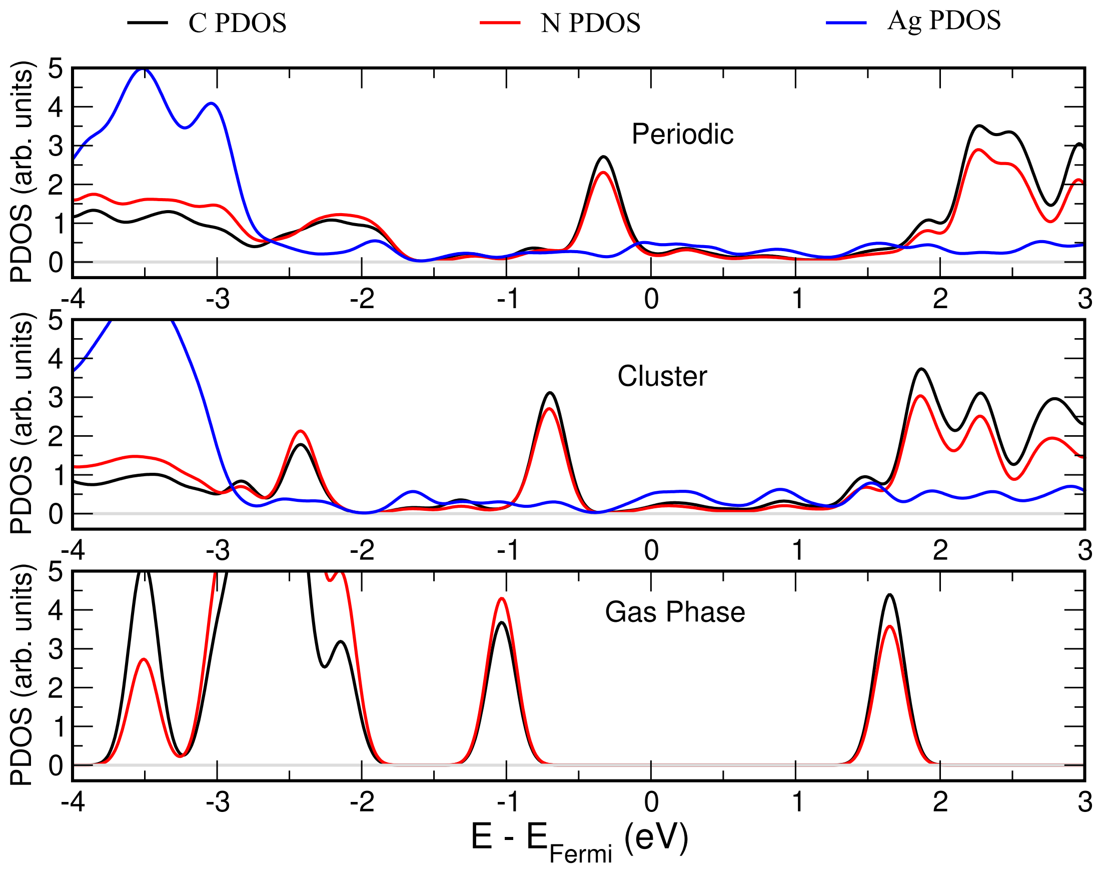

FIG. S2: Projected density of states of TCNE molecule adsorbed at Ag(100) periodic slab (top), TCNE molecule adsorbed at Ag(100) cluster (center), and TCNE molecule in the gas phase (bottom). Carbon, nitrogen and silver atomic PDOS are depicted by black, red and blue curves, respectively. A baseline depicted as a gray line has been added for clarity in all the panels.

In Fig. S4 we show the dependence of $\tilde{\Phi}^{\text{tip}}$ with respect to the kick strength. At all the considered positions below the the tip apex, a linear dependence is observed

We studied the dependence of the molecular induced dipole, μ^{ind}, with respect to the kick strength by performing TD-DFT simulations with a kick for a system composed of tipA and a benzene molecule at 4 Å below it. Fig. S5a shows the three different tip-molecule relative positions considered for this test. To isolate the molecular contribution from the much larger tip contribution, we computed μ^{ind} by integrating a region containing only the molecule (see Fig. S5b). We verified that in this region, the electronic density integrates to the number of electrons in the molecule. In Fig. S5, we show the dependence of μ^{ind} with respect to the kick

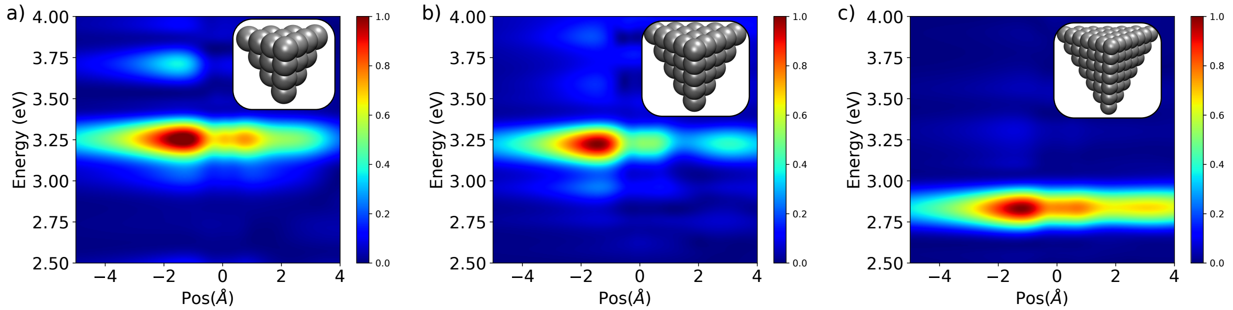

FIG. S3: Comparison $\tilde{\Phi}_{\text{tip}}$ for different tip sizes. a) Tip smaller than tip-A (20 Ag atoms) b) tip-A (35 Ag atoms) c) Tip bigger than tip-A (84 Ag atoms). The tip apex was set at the origin of coordinates as depicted in Figure 2a in the main text.

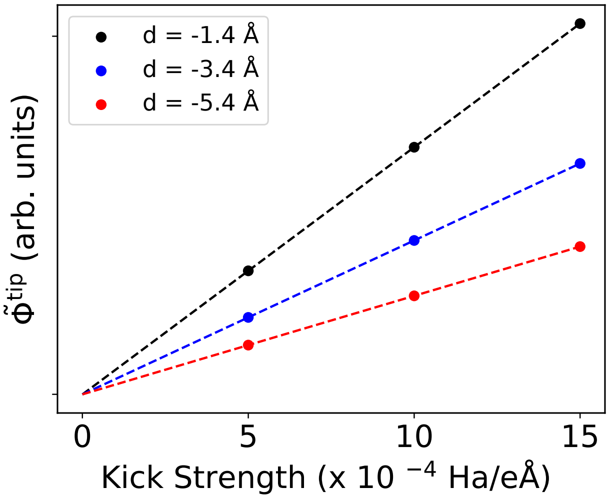

FIG. S4: Linearity of $\tilde{\Phi}^{\text{tip}}$ with respect to the kick strength. Dots corresponds to $\tilde{\Phi}^{\text{tip}}$ values at different distances, d, below the tip apex. Dashed lines are linear fits of the data points.

strength and find a linear dependence. This confirms that we are within the applicability realm of first-order perturbation theory. Moreover, the change of intensity of the induced dipole follows the same trend as the TERS image presented in the main text (see Fig3 g) with a maximum at d=2.5Å.

To analyze the shape of the local electromagnetic field, $\partial\tilde{\Phi}_{\text{tip}}/\partial z$, we fitted it by a Gaussian function defined as

$$f(x,y) = A_0 e^{-\left(\frac{1}{2\sigma_x^2}(x-x_0)^2+\frac{1}{2\sigma_y^2}(y-y_0)^2\right)} + B_0, \tag{25}$$

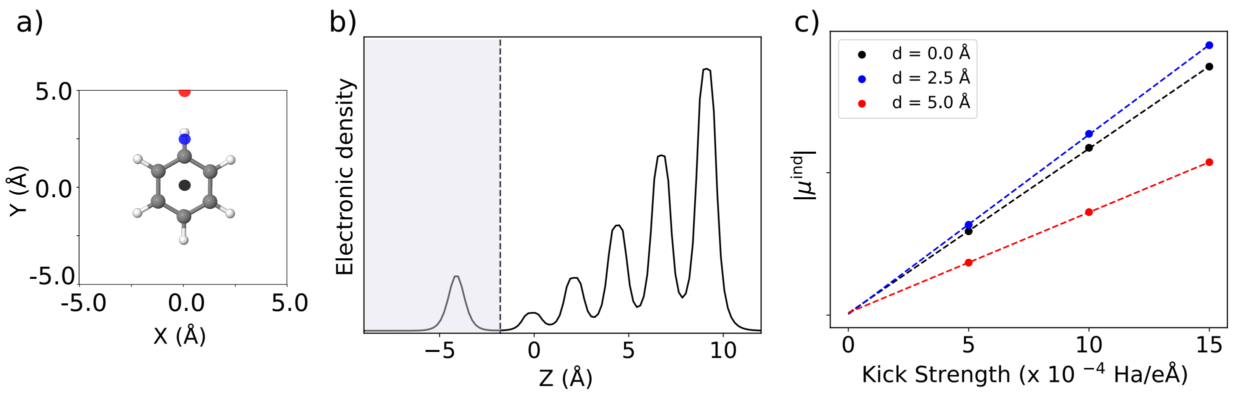

FIG. S5: a) Visual representation of the benzene molecule and the the 3 lateral positions of the tip apex considered for this test. b) Electronic density along the z-direction (main tip axis) integrated along the orthogonal xy plane. Molecular induced dipoles, μ^{ind}, were obtained by integrating the electronic density for $\infty < z < -1.8$ Å (gray area) c) Dependence of $|\mu^{\text{ind}}|$ at 532 nm with respect to the kick strength at different tip-molecule relative positions.

where (x_0, y_0) are the coordinates of maximum, and A_0 and B_0 are a normalization constant and an offset, respectively.

Fig. S6 and S7 show two-dimensional Gaussian fits of $\partial\tilde{\Phi}_{\text{tip}}/\partial z$ for tip-A model structure at 4 Å and 1.5 Å below the tip apex, respectively. On the former case, the Gaussian fit reproduces to some extent the reference TD-DFT data, but the fit presents a more moderate increase at its center and underestimates the maximum intensity by 20%. On the latter case, a Gaussian fit completely misses the rapid variation and sign change of local field observed in the reference data. In Fig. S8 and S9, we show analogous plots for the tip-B model structure. While a Gaussian fit for the data at 1.5 Å is clearly inadequate, at 4.0 Å the fit looks acceptable besides the fact that it cannot capture the radial asymmetry present in the reference data. We remark that in this work only tip-molecule distances greater than 4 Å have been considered.

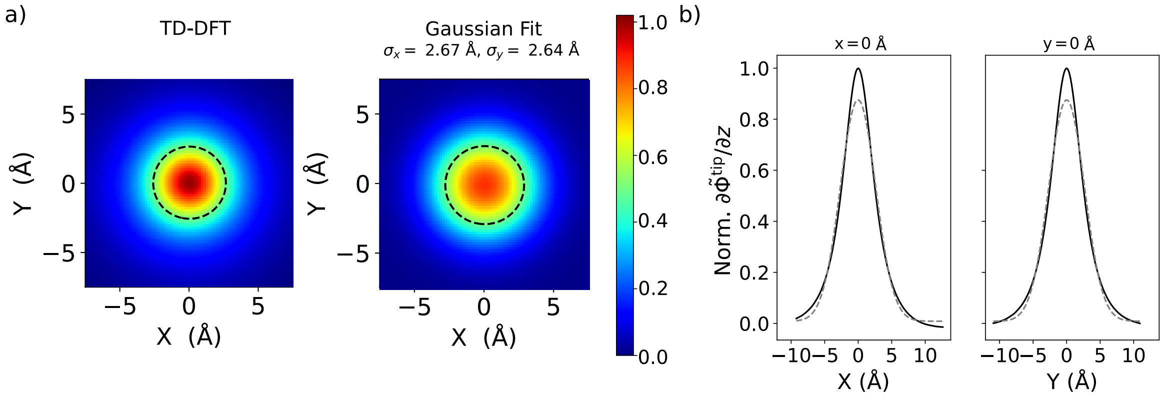

FIG. S6: Gaussian fit of a two-dimensional slice of $\partial\tilde{\Phi}_{\text{tip}}/\partial z$ for tip-A model structure at 4 Å below the tip apex. a) Normalized two-dimensional heat map. Dashed lines represent 0.5 isocontours. b) One dimensional cuts along x=0 Å and y=0 Å. Solid black line and gray dashed line represent the reference and Gaussian fit data, respectively. The tip apex was set at the origin of coordinates as depicted in Figure 2a in the main text.

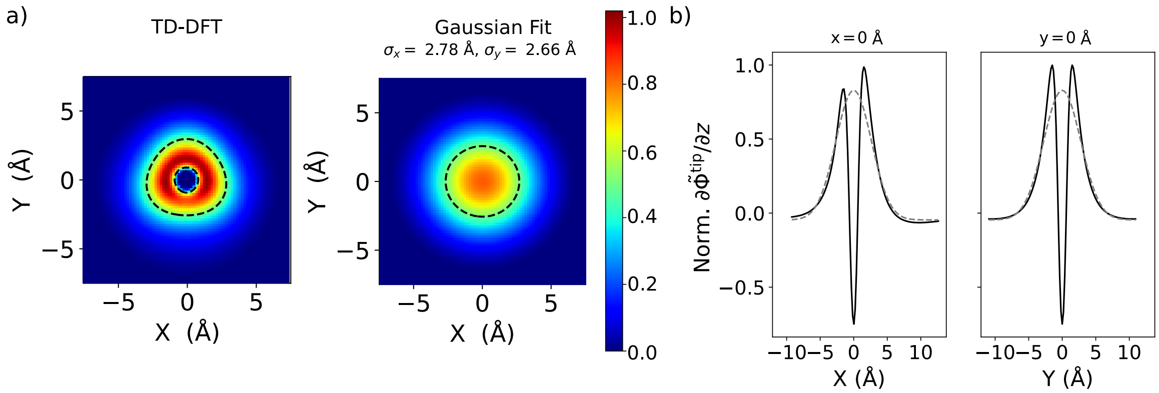

FIG. S7: Same as figure S6 for a slice taken at 1.5 Å below the tip apex.

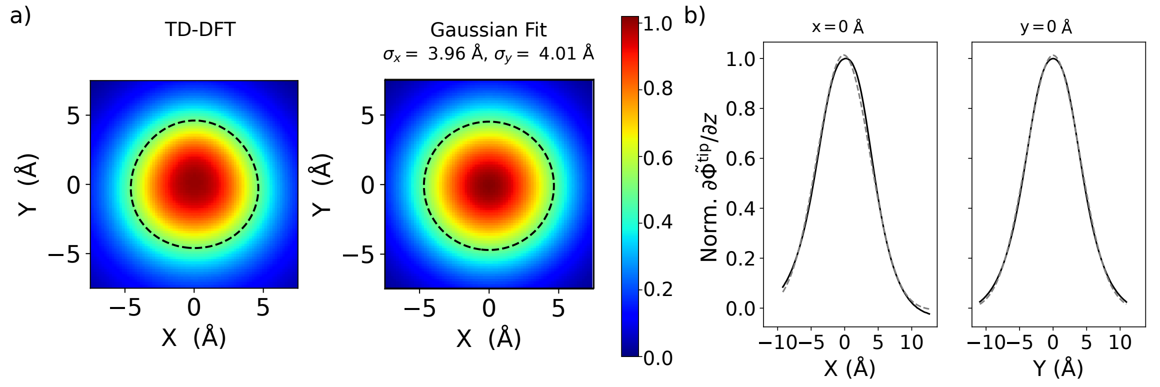

FIG. S8: Same as figure S6 for a tip-B model structure.

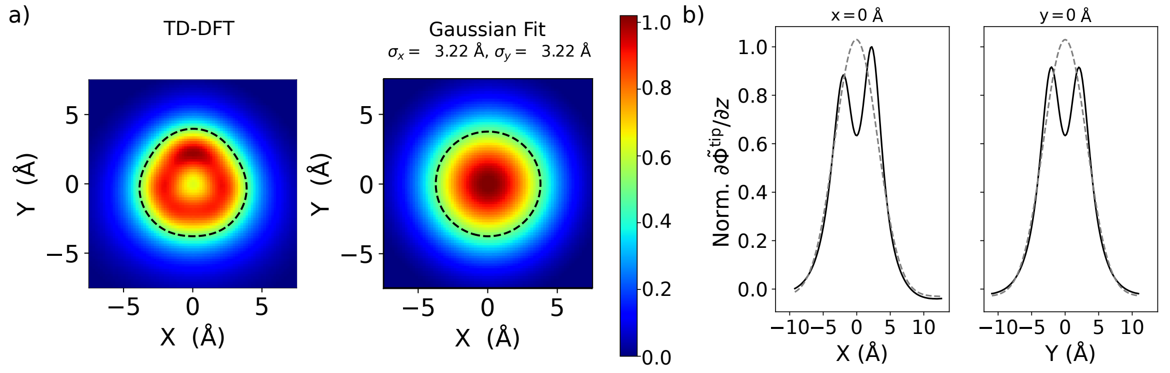

FIG. S9: Same as figure S7 for a tip-B model structure.

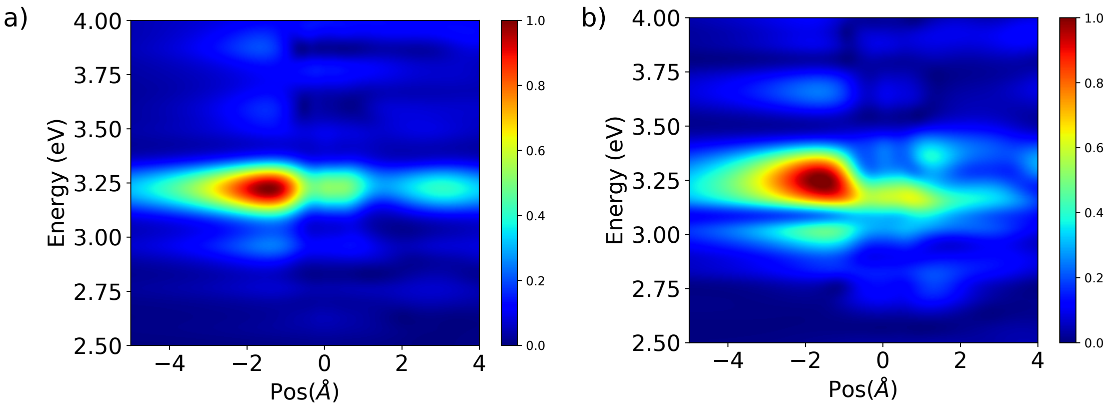

FIG. S10: Comparison of $\tilde{\Phi}_{\rm tip}$ of Tip-A computed using a) LDA and b) PBE exchange correlation functionals.

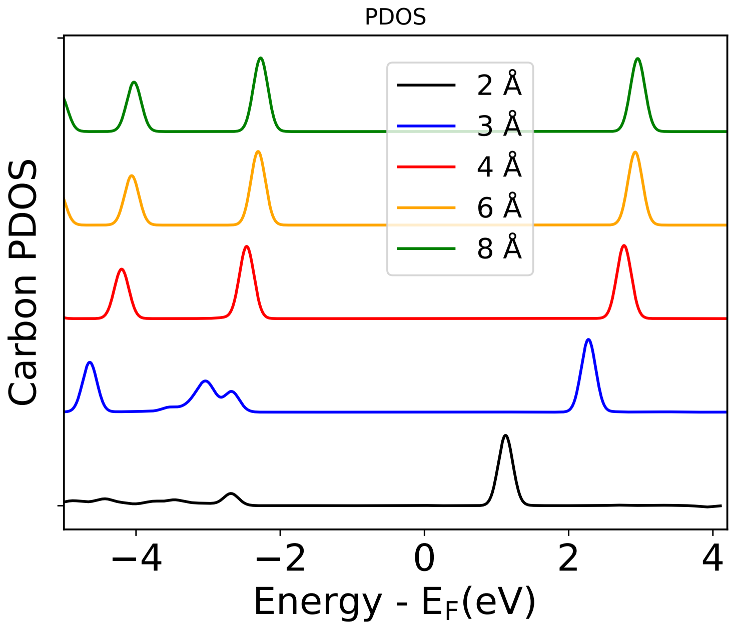

FIG. S11: Projected electronic density of states of benzene molecules as a function of molecule-tip distance.

IV. ADDITIONAL TERS IMAGES

In Fig. S12 and S14, we show further TERS images for the benzene and TCNE molecules, respectively.

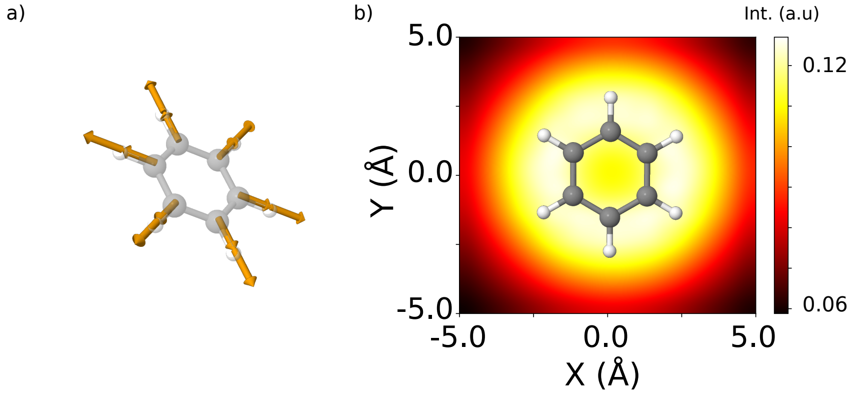

FIG. S12: TERS simulation of gas-phase benzene from local-field DFPT calculations. Normal mode displacements (a) and TERS images (b) of the the 1015 cm^{-1} (a_{1g}) mode for a molecule-tip apex distance of 4 Å.

To further verify the convergence of the TERS calculations with respect to the cluster size, we performed TERS simulations at some representative tip-molecule relative positions. In Fig. S13, we show the position dependence of the TERS signal along the molecular axis for six vibrational normal modes. In all cases, the results obtain with the small cluster are in semi-quantitative agreement with the ones obtained with the larger cluster.

V. COMPUTATIONAL SAVING OF THE PROPOSED METHOD

The reduced computational cost of the DFPT calculation with respect to the full real-time TDDFT simulations can be easily verified by comparing the two methods. However, as two different implementations are used for both methods, a completely fair, code-independent comparison is not possible at the moment. Instead, we state the cost of a typical calculation of each type with both codes, operating at optimal conditions of parallelization in CPUs. The timing for the TDDFT run (using the Octopus code) with a Dirac-delta perturbation to calculate the polarisability of a system comprising a silver tip A plus a benzene molecule (47 atoms) is of 324 core-hours (Intel Xeon IceLake-SP processors), in the Raven supercomputer

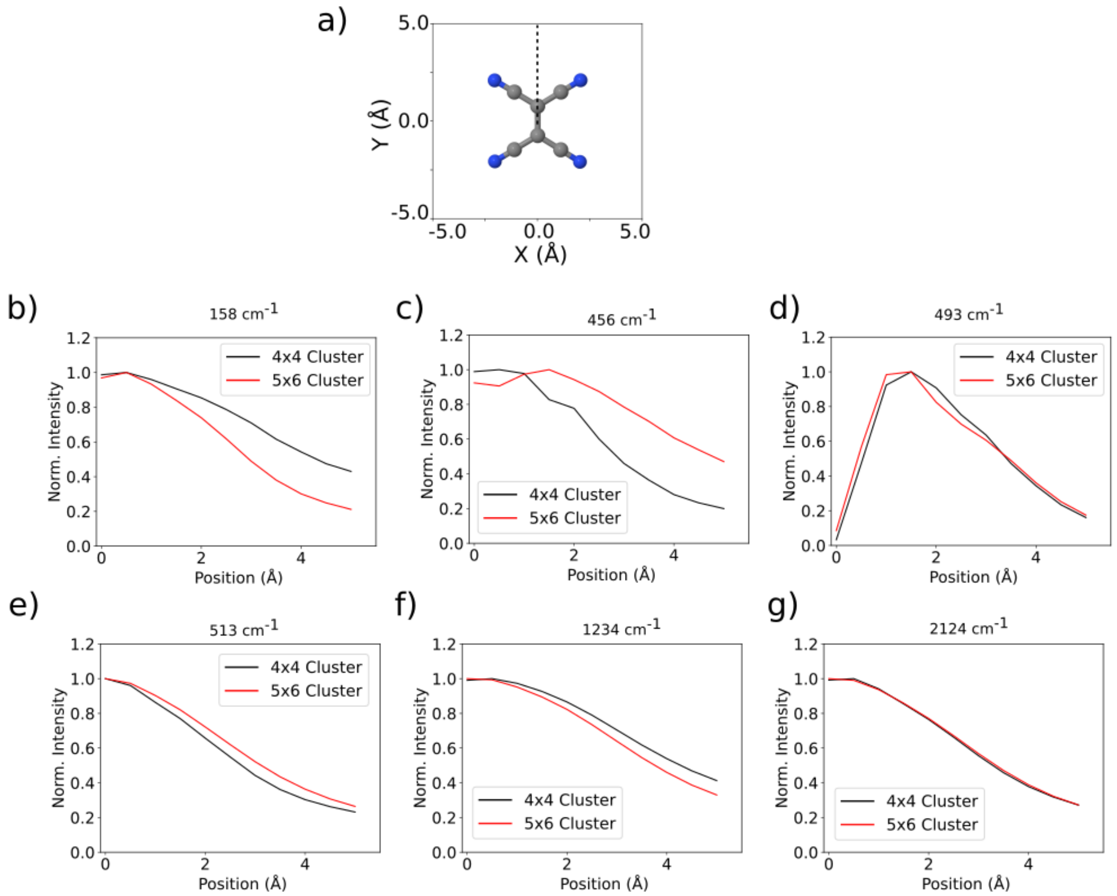

FIG. S13: a) Schematic depictions of the axis along which the TERS calculations were performed. b)-g) Normalized TERS intensity for selected vibrational modes.

installed at the Max-Planck Computing and Data Facility (https://docs.mpcdf.mpg.de). The computational cost of the DFPT calculations (using the FHI-aims code) on the equivalent system and machine is 0.02 core hours. While it is true that the DFPT calculation needs as input the Hartree potential obtained from the TDDFT run, the same potential can be used for any tip position, while each tip position demands a separate TDDFT calculation. The comparison of the cost demonstrates an approximately 15000-fold increase in computational efficiency, which will become larger for tip models containing more metal atoms. Furthermore, while the DFPT calculations have a $N \log(N)$ scaling with respect to the number of atoms (N) up to systems with an $\approx$ size of around 1000, the TDDFT implementation scales as N^3.

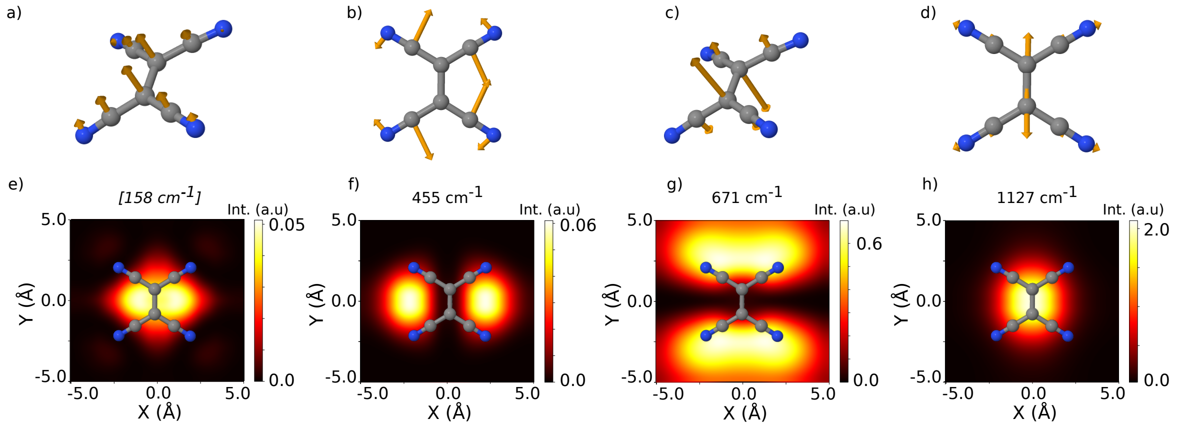

FIG. S14: Simulated TERS images of TCNE in isolation, but at the adsorbed geometry with the addition of 1 electron to the molecule without further geometry relaxation (TCNEads-1e). a), b), c) and d) Normal mode displacements of selected vibrational modes of TCNE@Ag(100). The surface has been deleted for clarity. e), f), g) and h) TERS images of the depicted normal modes for TCNEads. In all cases a molecule-apex distance of 4 Å was employed. Frequency within square brackets in panel e) denotes the lack of an equivalent normal mode eigenvector in the TCNEads1e calculation.

REFERENCES

[1] V. Blum, R. Gehrke, F. Hanke, P. Havu, V. Havu, X. Ren, K. Reuter, and M. Scheffler, Comp. Phys. Comm. **180**, 2175 (2009).

[2] J. P. Perdew and Y. Wang, Phys. Rev. B **45**, 13244 (1992).

[3] V. G. Ruiz, W. Liu, E. Zojer, M. Scheffler, and A. Tkatchenko, Phys. Rev. Lett. **108**, 146103 (2012).

[4] A. H. Larsen, J. J. Mortensen, J. Blomqvist, I. E. Castelli, R. Christensen, M. Dułak, J. Friis, M. N. Groves, B. Hammer, C. Hargus, E. D. Hermes, P. C. Jennings, P. B. Jensen, J. Kermode, J. R. Kitchin, E. L. Kolsbjerg, J. Kubal, K. Kaasbjerg, S. Lysgaard, J. B. Maronsson, T. Maxson, T. Olsen, L. Pastewka, A. Peterson, C. Rostgaard, J. Schiøtz, O. Schütt, M. Strange, K. S. Thygesen, T. Vegge, L. Vilhelmsen, M. Walter, Z. Zeng, and K. W. Jacobsen, J. Phys.: Condens. Matter **29**, 273002 (2017).

[5] X. Andrade, D. Strubbe, U. De Giovannini, A. H. Larsen, M. J. T. Oliveira, J. Alberdi-Rodriguez, A. Varas, I. Theophilou, N. Helbig, M. J. Verstraete, L. Stella, F. Nogueira, A. Aspuru-Guzik, A. Castro, M. A. L. Marques, and A. Rubio, Phys. Chem. Chem. Phys. **17**, 31371 (2015).

[6] N. Tancogne-Dejean, M. J. T. Oliveira, X. Andrade, H. Appel, C. H. Borca, G. Le Breton, F. Buchholz, A. Castro, S. Corni, A. A. Correa, U. De Giovannini, A. Delgado, F. G. Eich, J. Flick, G. Gil, A. Gomez, N. Helbig, H. Hübener, R. Jestädt, J. Jornet-Somoza, A. H. Larsen, I. V. Lebedeva, M. Lüders, M. A. L. Marques, S. T. Ohlmann, S. Pipolo, M. Rampp, C. A. Rozzi, D. A. Strubbe, S. A. Sato, C. Schäfer, I. Theophilou, A. Welden, and A. Rubio, J. Chem. Phys. **152**, 124119 (2020).

[7] O. A. Douglas-Gallardo, M. Berdakin, T. Frauenheim, and C. G. Sánchez, Nanoscale **11**, 8604 (2019).
